# Methyladenine as a regulator of biomolecular condensation

**DOI:** 10.3389/fmolb.2025.1722147

**Published:** 2025-11-26

**Authors:** Roberto Arsiè, Gian G. Tartaglia, R. Martin Vabulas

**Affiliations:** 1 Charité-Universitätsmedizin Berlin, Institute of Biochemistry, Berlin, Germany; 2 Centre for Human Technologies (CHT), RNA System Biology Lab, Istituto Italiano di Technologia, Genoa, Italy

**Keywords:** N1-methyladenine, N6-methyladenine, RNA-binding proteins, granulation, protein aggregation

## Abstract

Ribonucleotide modifications modulate RNA structure and stability, thereby influencing RNA turnover and a wide range of cellular functions. Recent studies have revealed that specific RNA modifications can also affect the formation, composition, and material properties of biomolecular condensates. This review explores how N1-methyladenine (m^1^A) and N6-methyladenine (m^6^A) contribute to RNA-driven phase transitions and the balance between adaptive granulation and pathological protein aggregation. m^1^A can act as a protective tag: by altering local RNA structure and RNA-protein interactions, it promotes the sequestration of selected transcripts into dynamic stress granules and facilitates the resumption of protein synthesis after stress. During chronic proteotoxicity, m^1^A helps prevent aberrant RNA-protein entanglement. However, when present on pathogenic repeat RNAs, m^1^A can recruit aggregation-prone proteins and exacerbate pathology. On the other hand, m^6^A functions as both a structural switch and a multivalent docking signal. Multiple m^6^A sites enhance the binding of cognate reader proteins to a transcript, thereby accelerating stress granule assembly. m^6^A modification has also been implicated in organizing nuclear condensates such as HSATIII lncRNA assemblies. We discuss mechanistic models that aim to reconcile the diverse roles of methyladenine, highlight current experimental challenges, and outline emerging approaches for addressing the remaining open questions.

## 1 Introduction

Eukaryotic cells employ diverse strategies to achieve spatial and temporal organization. Under specific biochemical conditions, subsets of cellular proteins and nucleic acids undergo liquid-liquid phase separation (LLPS) from the surrounding cytoplasm to form biomolecular condensates (Zacco et al., 2024). These micron-scale assemblies are integral component of eukaryotic cell organization, regulating key processes such as stress adaptation and RNA metabolism (Banani et al., 2017; Shin and Brangwynne, 2017; Sabari et al., 2020; Alberti and Hyman, 2021; Gorsheneva et al., 2024). Unlike membrane-bound organelles, biocondensates are dynamic entities that can assemble and dissemble reversibly in response to environment cues. Their formation depends on the intrinsic biophysical properties of constituent proteins and RNAs, which drive demixing from the surrounding aqueous milieu. These biocondensates function as subcellular hubs that concentrate specific polypeptides and transcripts (Hirose et al., 2023), thereby coordinating numerous cellular processes in a spatiotemporally-controlled, membrane-independent manner. Stress granules (SG) and processing bodies (PB) are two examples of cytosolic ribonucleoprotein assemblies formed by LLPS under physiological conditions or in response to stressors such as heat shock and arsenite exposure (Luo et al., 2018; Protter et al., 2018). In the nucleus, nuclear stress bodies (nSB) represent analogous membraneless organelles that assemble upon thermal stress.

The assembly of biocondensates depends on weak, multivalent interactions among their molecular constituents. In particular, proteins containing intrinsically disordered regions (IDR) or low-complexity domains (LCD) are especially prone to form granular structures, as they serve as scaffolds for intra- and intermolecular interactions (Shin and Brangwynne, 2017; Martin and Mittag, 2018; Vodnala et al., 2021). RNA also plays a central role in biomolecular condensate formation: as a flexible and multivalent polymer, it can engage in numerous interactions with diverse proteins and other RNA molecules (Zhang et al., 2015; Saha et al., 2016; Roden and Gladfelter, 2021; Ripin and Parker, 2023). This intricate network of dynamic interactions enables cells to rapidly assemble and disassemble condensates in response to changing physiological conditions (Cairo et al., 2024; Zhang et al., 2025).

While dynamic and reversible LLPS is essential for cellular homeostasis, its dysregulation can lead to pathological consequences (Zacco et al., 2024). A critical distinction exists between the reversible formation of liquid-like condensates and their conversion into static, irreversible fibrillar aggregates - a hallmark of numerous diseases (Molliex et al., 2015; Alberti and Dormann, 2019; Mathieu et al., 2020). For example, RNA-binding proteins such as TDP-43 and FUS normally form dynamic liquid droplets, but in neurodegenerative conditions like amyotrophic lateral sclerosis (ALS) and frontotemporal dementia (FTD), they accumulate as insoluble fibrillar inclusions (Patel et al., 2015; Carey and Guo, 2022). Therefore, understanding the molecular switches that govern these liquid-to-solid transitions may be crucial for developing potential therapeutic strategies.

Among the various biochemical and environmental factors that drive biocondensate formation, a growing body of evidence indicates the role of certain RNA modifications, specifically, N6-methyladenine (m^6^A) and N1-mehtyladenine (m^1^A). These modifications are installed by specific enzymes (“writers”), recognized by cognate “readers”, and removed by dedicated demethylases (“erasers”) - a regulatory machinery that enables dynamic adjustment of methyladenine abundance and its functional activity.

## 2 Methods for m^1^A and m^6^A detection

A wide range of methods have been developed to detect and quantify RNA modifications. One common approach relies on commercially available antibodies that recognize m^1^A and m^6^A in RNAs. Typically, these antibodies are used to enrich transcripts carrying the modification, after which the immunoprecipitated RNAs are subjected to next-generation sequencing to map the modified sites (Dominissini et al., 2012; 2016; Meyer et al., 2012; Li et al., 2016). These approaches enable transcriptome-wide mapping of the selected modification but suffer from low resolution (approximately 100 base pairs). Inevitably, the results are influenced by antibody specificity and cross-reactivity (Schwartz, 2018; Grozhik et al., 2019). Cross-linking or reverse-transcription-based variants inducing mutational signatures increase the resolution, but still rely on immunoprecipitation (Linder et al., 2015; Li et al., 2017; Safra et al., 2017; Grozhik et al., 2019; Koh et al., 2019; Roberts et al., 2021).

Chemical or enzymatic conversion approaches do not require antibodies. These methods exploit the fact that certain RNA modifications alter the activity of nucleases, deaminases, or reverse transcriptases, thereby generating characteristic sequence “signatures” that can be detected through high-throughput sequencing. For m^6^A detection, several antibody-independent protocols have been developed, such as MAZTER-seq, in which the activity of the endoribonuclease MazF is blocked by m^6^A (Garcia-Campos et al., 2019), and DART-seq, where an engineered APOBEC enzyme edits nucleotides near m^6^A sites (Meyer, 2019). These methods provide improved resolution without the need for antibodies; however, they require engineered enzymes and yield motif-restricted results. For m^1^A detection, methods take advantage of its ability to affect the fidelity of reverse transcription, leading to characteristic mutation signatures (Zhou et al., 2019). A major challenge, however, lies in reliably detecting these misincorporation events (Sas-Chen and Schwartz, 2019). Controls often include AlkB-mediated demethylation or the Dimroth rearrangement. The latter–chemical conversion of m^1^A to m^6^A under alkaline and heat conditions - poses a significant analytical challenge for accurate RNA modification mapping and quantification. If the rearrangement occurs inadvertently during RNA handling or library preparation, m^1^A residues may be misclassified as m^6^A. This artifact is particularly problematic for antibody-based m^6^A mapping approaches, which cannot distinguish between endogenous m^6^A and rearranged m^1^A. As a result, false-positive m^6^A sites may be reported, distorting modification maps and complicating downstream biological interpretation.

Thin-layer chromatography (TLC) and liquid chromatography-tandem mass spectrometry (LC-MS/MS) remain the gold standards for global quantification of RNA modifications (Grosjean et al., 2004; Herbert et al., 2024). These approaches provide absolute stoichiometries and enable the simultaneous detection of multiple modifications. However, they offer little or no positional information and require relatively large amounts of input RNA, making them well suited for studying abundant RNA species (Taoka et al., 2018; Suzuki et al., 2020).

Both m^6^A and m^1^A can, in principle, be detected through alterations in the ionic current during Nanopore-direct RNA sequencing (Leger et al., 2021). When combined with machine learning-based models, this approach enables antibody-free, single-molecule detection. While promising, the relatively low signal-to-noise ratio remains a major challenge, and extensive validation is still required.

## 3 Computational approaches for mapping and predicting m^1^A and m^6^A

Alongside experimental mapping, bioinformatics has become central to detecting and interpreting RNA methylation marks. For both m^6^A and m^1^A, antibody-based enrichment methods (MeRIP-seq, m^1^A-seq) generate peaks that require dedicated computational pipelines for analysis. Peak callers such as exomePeak and MACS2 have been widely used to analyse m^6^A data (Dominissini et al., 2012; Meyer et al., 2012; Linder et al., 2015), while improved frameworks such as exomePeak2 and MeTPeak provide bias correction (Cao et al., 2014; Cui et al., 2016) and statistical modeling. For m^1^A, computational workflows must also account for potential Dimroth rearrangement to m^6^A, which complicates the interpretation of sequencing data (Safra et al., 2017; Zhou et al., 2019). Chemical conversion or mutational signature-based approaches provide single-nucleotide resolution but yield heterogeneous misincorporation patterns that require careful computational filtering (Grozhik et al., 2019). More recently, direct RNA Nanopore sequencing combined with machine learning-based classifiers has enabled antibody-free identification of m^6^A and m^1^A at single-molecule resolution (Leger et al., 2021). Such computational frameworks are essential for distinguishing true modification signatures from sequencing noise and for integrating orthogonal validation data, such as LC–MS/MS measurements.

In parallel, machine learning and modeling tools can be leveraged to predict methylation sites *de novo* and assess their structural and functional impacts. For m^6^A, tools such as SRAMP (Zhou Y. et al., 2016) and DeepM6ASeq (Zhang and Hamada, 2018) combine sequence motifs like DRACH with structural context (e.g., predicted accessibility, pairing propensity). On the m^1^A front, iRNA-3typeA (Chen et al., 2018) and ISGm1A (Liu et al., 2020) use feature sets derived from k-mer frequencies, nucleotide physicochemical properties, and secondary structure proxies to score candidate adenines. To capture how modifications may alter structure or binding energetics, computational tools such as *ViennaRNA* have been extended to support folding analyses that include parameters for modified bases, allowing comparison of RNAs containing m^6^A or m^1^A (Varenyk et al., 2023). Additionally, *catRAPID* has incorporated m^6^A information to estimate how this modification changes RNA–protein binding propensities (Vandelli et al., 2024). Together, these computational tools enable researchers to move from raw sequencing reads to predicted methylomes, and from there to hypotheses about how m^6^A and m^1^A influence RNA folding, protein binding, and ultimately condensate dynamics.

## 4 m^6^A as a phase separation enhancer

N6-methyladenine (m^6^A) is the most abundant modification found on eukaryotic mRNA (Desrosiers et al., 1974; Dubin and Taylor, 1975). m^6^A is installed on RNAs by a multi-protein methyltransferase complex: the catalytic METTL3 subunit pairs with METTL14 to form a stable heterodimer, and WTAP targets this complex to RNAs (Liu et al., 2014; Wang et al., 2016). The m^6^A mark is reversible. FTO (fat mass and obesity-associated protein) was the first enzyme shown to demethylate m^6^A (Jia et al., 2011), and ALKBH5 later was identified as a second m^6^A demethylase affecting mRNA export and metabolism in mouse germ cells (Zheng et al., 2013). Together, these erasers make m^6^A a dynamic and reversible modification. m^6^A is recognized by specific reader proteins, most notably member of the YTH-domain family, such as YTHDF1-3 and YTHDC1 (Zhu et al., 2014; Zaccara and Jaffrey, 2020). Biochemically, m^6^A alters RNA duplex stability and can locally “open” RNA structure, favoring a single-strand conformation, which could influence binding of other factors (Liu et al., 2015; Roost et al., 2015). Functionally, m^6^A impacts nearly every step of mRNA metabolism. Early transcriptome-wide maps revealed that m^6^A is enriched near stop codons and within long internal exons, where it influences RNA splicing (Dominissini et al., 2012). Binding of nuclear m^6^A-marked pre-mRNAs by YTHDC1 was also shown to modulate splicing (Xiao et al., 2016). YTHDF2 binding to m^6^A generally decreases mRNA stability, thereby shortening the half-life of its targets (Wang et al., 2014; Du et al., 2016). In contrast, YTHDF1 binds m^6^A-modified mRNAs to enhance their translation efficiency (Wang et al., 2015).

**FIGURE 1:**
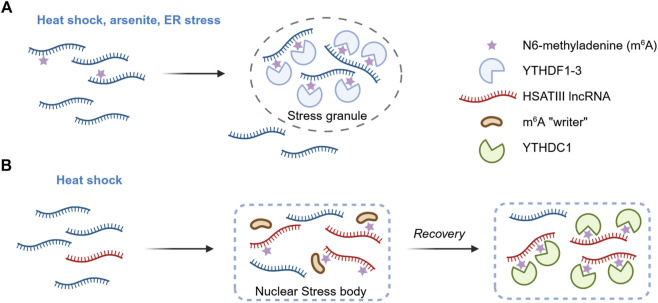
m^6^A as a regulator of biomolecular condensation. **(A)** m^6^A influences stress granule formation. In response to stress, the assembly of stress granules is promoted by multiple m^6^A modifications on RNAs and the subsequent recruitment of the reader proteins YTHDF1-3. Length-dependent transcript enrichment in SG is also mediated by m^6^A. However, the contribution of m^6^A to stress granule dynamics appears to be context-dependent, with some studies reporting a strong effect on granule assembly and others indicating only a modest influence (not shown). **(B)** m^6^A in nuclear stress bodies. Following heat shock, the pericentromeric lncRNA HSATIII is transcribed and drives the assembly of nuclear stress bodies. Components of the m^6^A methyltransferase complex are recruited, leading to post-transcriptional modification of HSATIII. Upon stress recovery, the m^6^A reader YTHDC1 is sequestered at nuclear stress bodies, leading to its transient depletion from the surrounding nucleoplasm and altering the splicing patterns of target pre-mRNAs.

Recent studies have shown that m^6^A modifications on mRNAs can enhance their capacity for phase separation, together with their associated m^6^A reader proteins. For example, Ignatova’s research group demonstrated that during oxidative stress induced by arsenite exposure, the total m^6^A level increased in U2OS cells, with approximately 8% more mRNAs found to be methylated (Anders et al., 2018). The modification colocalized with stress granules also upon heat shock. During stress, the post-transcriptional methylation was enriched in the 5′ UTRs of mRNAs. The m^6^A reader YTHDF3 bound to these stress-modified RNAs and relocated with them to stress granules. Removed from the translating pool and triaged into stress granules, m^6^A-labelled mRNAs seem to be dynamically regulated (Figure 1A).

Several studies have further demonstrated that the m^6^A reader proteins YTHDF1, YTHDF2, and YTHDF3 undergo liquid-liquid phase separation (LLPS) both *in vitro* and *in vivo*, and that this process is markedly enhanced in the presence of m^6^A-modified RNA (Gao et al., 2019; Ries et al., 2019; Fu and Zhuang, 2020). Multivalence is critical, as multiple m^6^A modifications were required to cluster sufficient YTHDF molecules to nucleate condensates. Various environmental stressors - such as heat shock, arsenite exposure, endoplasmic reticulum stress - promoted the relocalization of these proteins and the associated m^6^A-containing RNAs from the cytosol to stress granules. Interestingly, knockout of METTL14 did not affect the formation of stress granules or P-bodies, but the redistribution of YTHDF2 within these compartments was greatly reduced (Ries et al., 2019). This finding indicates that m^6^A reader proteins must bind modified RNAs in order to move to membraneless compartments (Figure 1A). The LLPS of YTHDF2 was enhanced by m^6^A under stressful conditions (Man et al., 2025), whereas mutations in its m^6^A-binding domain impaired granule formation *in vivo* (Wang et al., 2020). Noteably, m^6^A has also been shown to govern the preferential enrichment of long mRNAs within stress granules (Ries et al., 2023).

These findings were supported by another study in which the authors implemented a FRET-based method to track m^6^A-modified RNAs and their interactions with reader proteins (Li et al., 2024). They showed that m^6^A-marked transcripts rapidly accumulated in stress granules during stress and relocated back to the cytosol during the recovery phase. Knockdown of YTHDF2 accelerated granule disassembly, enabling faster mRNA release and translation recovery.

Challenging the findings described above, the laboratory of Roy Parker questioned the functional significance of m^6^A in stress granules formation (Khong et al., 2022). In their study, knockout of the m^6^A writer METTL3 did not alter the recruitment of several poly-m^6^A-modified mRNAs to arsenite induced stress granules, with m^6^A status explaining only 6% of the variance in RNA partitioning into these granules. Furthermore, in an artificial system designed to enhance reader protein interaction, 25 copies of YTHDF were tethered to a single transcript, yet no significant increase in RNA recruitment to granules was observed. Aside from the non-physiological nature of tethering 25 proteins to one transcript and correlations drawn from datasets across different studies and cell lines, the apparent discrepancy may also reflect dependencies on stress conditions, cell-types specificities, or yet-to-be-identified compensatory mechanisms.

m^6^A has also recently been implicated in the regulation of membraneless organelle in the nucleus. During thermal stress, the long non-coding RNA (lncRNA) HSATIII is expressed and contributes to the formation of nuclear stress bodies (nSB) (Jolly et al., 2004; Biamonti and Vourc’h, 2010). Ninomiya and colleagues showed that HSTAIII sequence is highly m^6^A-modified and that this modification sequestered m^6^A regulatory factors into the condensate (Ninomiya et al., 2021). Using comprehensive RNA-protein interaction proteomics (m^6^A-RIP-seq and LC-MS/MS on purified HSATIII fragments), the authors found that approximately 11% of the first adenine in the GGAAU repeat unit of HSATIII is methylated at position 6, and that the m^6^A writer complex is recruited into nSBs during stress recovery. Furthermore, m^6^A-modified HSATIII sequestered the nuclear m^6^A reader YTHDC1 within nSBs during stress recovery, thereby lowering its nucleoplasmic concentration. Since YTHDC1 is known to promote m^6^A-dependent splicing, its depletion lead to increased intron retention in a subset of m^6^A-marked pre-mRNAs. This work therefore unveiled a mechanistic link between an m^6^A-modified structural lncRNA, biocondensation, and genome-wide regulation of temperature-dependent splicing. HSATIII appears to act both as a “reaction crucible” that promotes local biochemical events and as a “molecular sponge” that titrates m^6^A machinery proteins (Figure 1B).

## 5 m^1^A in stress granules and protein aggregates

N1-methyladenine (m^1^A) was first discovered in non-coding RNA in the 1960s, specifically at position 58 of tRNAs and within the large subunit of ribosomal RNA (Dunn, 1961; Shima and Igarashi, 2020). This post-transcriptional modification is conserved across Bacteria, Archaea and Eukaryotes. In eukaryotes, m^1^A is installed by several methyltransferases. The TRMT6-TRMT61A complex acts in the cytoplasm, modifying both tRNAs and a subset of mRNAs that contain a conserved GUUCRA motif reminiscent of the tRNA TΨC loop. The homologous enzymes TRMT61B and TRMT10C are active in mitochondria and modify mitochondrial tRNAs and rRNAs there (Chujo and Suzuki, 2012; Vilardo et al., 2012; Bar-Yaacov et al., 2016). In the nucleolus, RRP8/NML creates the m^1^A1322 mark in 28S rRNA (Waku et al., 2016). m^1^A is a reversible modification: mammalian FTO and the AlkB homologs ALKBH1, ALKBH3 can demethylate m^1^A in RNA indicating dynamic regulation. Among these, ALKBH3 is the main demethylase acting on mRNAs.

**FIGURE 2:**
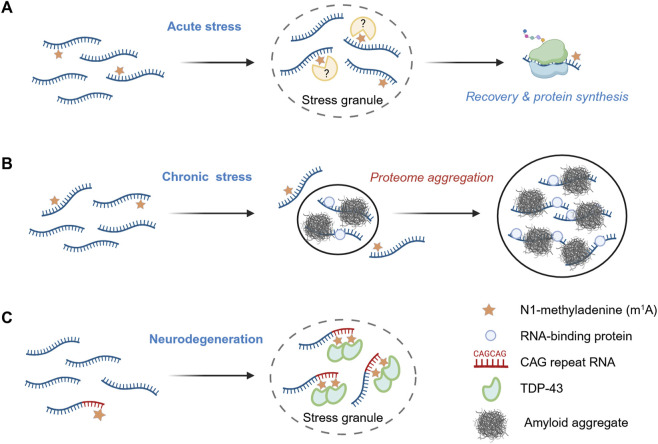
m^1^A as a regulator of biomolecular condensation. **(A)** Acute stress response. During acute stress, m^1^A-modified mRNA are preferentially sequestered into stress granules, and, upon stress release, are translated more efficiently than their non-methylated counterparts. **(B)** Chronic stress conditions. Under prolonged stress, such as during amyloidogenesis, reduced m^1^A levels promote protein aggregation and enhance the recruitment of bystander RNA-binding proteins. **(C)** m^1^A -modified CAG repeat RNAs and TDP-43 recruitment. Ectopic expression of RNAs containing expanded CAG repeats promotes stress granule assembly and leads to extensive m^1^A modification. This modification facilitates the relocalization of the RNA-binding protein TDP-43 from the nucleus to cytosolic stress granules, contributing to aberrant aggregation events associated with neurodegeneration.

Structurally, the addition of a methyl group at position 1 on the adenine base disrupts its ability to form standard hydrogen bonds and imparts a positive charge on it, if at physiological pH. This alteration affects the secondary and tertiary structures of the modified RNA, strongly influencing its intra- and intermolecular interactions. In mRNA, m^1^A exerts context- and position-dependent effects on transcripts’ translation and stability. One of the first m^1^A mapping studies revealed that m^1^A in 5′UTRs, especially in the proximity to the cap, correlates with increased translation efficiency (Li et al., 2017). In contrast, the same study showed that m^1^A within coding sequences tends to inhibit translation, likely by blocking codon-anticodon pairing. Several m^1^A “readers” have been identified among cytoplasmic YTH domain-containing proteins. For instance, YTHDF3 was found to specifically bind m^1^A sites in IGF1R mRNA and recruit RNA decay machinery, thereby downregulating that transcript (Zheng et al., 2020). Thus, m^1^A appears to function as a molecular switch that can either promote translation (when located in 5′UTR) or mark transcripts for degradation (through YTHDF binding), linking methylation to mRNA stability, localization, and translation control.

m^1^A is also a stress-related RNA modification, and growing evidence suggests its involvement in biomolecular condensation. A recent analysis uncovered that stress granule (SG)-enriched transcripts are statistically more likely to contain the m^1^A consensus motif GUUCRA, indicating a function link between m^1^A and granule localization (Alriquet et al., 2020). The study further showed that the m^1^A-installing TRMT6/TRMT61A complex accumulates in stress granules upon heat shock or arsenite treatment in HeLa cells. LC-MS/MS analyses demonstrated that SG-associated RNA contains significantly higher amounts of m^1^A compared to bulk cytosolic mRNA. The authors engineered a reporter mRNA harboring a single m^1^A motif in the 5′UTR and compared it with a non-methylatable mutant. Under normal conditions, both transcripts exhibited comparable translation efficiency. However, during heat shock, translation of the wild-type reporter was repressed more rapidly than that of the control. Upon recovery, translation from the m^1^A-containing reporter resumed more quickly, producing substantially higher protein levels than the non-methylatable counterpart (Figure 2A).

These results indicate an active safeguarding role of m^1^A during acute stress. Subsequent work extended this protective function to conditions of chronic proteotoxic stress, suggesting a general role of m^1^A in maintaining cellular homeostasis (Alriquet et al., 2025). Using a reporter system similar to that described above, the authors demonstrated that protein translation from methylation-deficient transcripts is impaired during amyloidogenesis. Forced reduction of m^1^A tagging–either by knockdown of the methyltransferase subunit TRMT61A or by orthogonal upregulation of the m^1^A demethylase ALKBH3 - led to increased amyloidogenesis in HeLa cells. Proteomic analysis of aggregates from m^1^A-deficient cells revealed a threefold increase in the co-aggregation of amyloid with bystander proteins, particularly with RNA-binding proteins (RBPs). Amyloid was more strongly associated with mRNA under these conditions. Together, these findings support a model in which m^1^A protects transcripts from entanglement with misfolded proteins, thereby limiting the RNA-driven propagation of protein aggregation during amyloidogenesis (Figure 2B).

A recent study by the Wang laboratory uncovered specific circumstance in which m^1^A can have the opposite effect, enhancing protein aggregation (Sun et al., 2023). The authors observed an accumulation of m^1^A in ectopically expressed CAG repeat RNAs, which are characteristic of several hereditary neurodegenerative disorders, including Huntington’s disease, spinocerebellar ataxia, and amyotrophic lateral sclerosis. Longer CAG tracts resulted in a higher level of adenine methylation and correlated with lower expression of the demethylase ALKBH3, suggesting a potential role of N1-methyladenine in the pathogenesis of triplet-repeat expansion diseases. Elevated m^1^A levels were also associated with enhanced neurodegeneration in *C. elegans* and *Drosophila*. The authors showed that m^1^A is recognized by the two RNA recognition motif (RRM) domains of TDP-43, a protein known to mislocalize and aggregate in the cytosol during neurodegeneration (Dai et al., 2018). Consistently, the authors observed the mislocalization and aggregation of TDP-43 upon ectopic expression of CAG repeat RNA in U2OS cells. In the cytosol, TDP-43 and CAG repeat RNAs co-localized with the stress granule marker G3BP1, and this association was reduced upon ALKBH3 overexpression (Figure 2C). Long CAG repeats with multiple m^1^A modifications altered the biophysical properties of TDP-43 condensates, shifting their phase behavior toward less soluble, amyloid-like aggregates. These findings highlight the toxic potential of m^1^A as a pathological enhancer of RNA-protein condensation, whereby this RNA modification is hijacked by expanded CAG repeats to promote aggregate formation and neurodegeneration. Gaining a precise mechanistic understanding of how m^1^A exerts protective versus toxic effects will require experimental advances to identify the structural changes that precede these divergent cellular outcomes.

## 6 Discussion

It seems surprising that the addition of a single methyl group can so strongly influence the intracellular behavior of a much larger mRNA molecule. At least two, potentially interconnected, explanations can be proposed: (1) methylation-induced alterations in the global RNA architecture, and (2) localized changes in the bonding capacity of methyladenine. As noted previously, the addition of a methyl group at the N1 position of adenine imparts a positive charge and prevents both Watson–Crick and Hoogsteen base pairing, thereby profoundly influencing intra- and intermolecular associations as well as the formation of structural motifs such as RNA hairpins (Micura et al., 2001; Zhou H. et al., 2016). These structural perturbations may diminish an mRNA’s ability to engage stably with RNA-binding proteins or other RNAs. This view is consistent with the accumulating evidence that nucleic acid structure can modulate, and in some cases promote, protein aggregation both *in vitro* and *in vivo*.

Despite significant recent advances in the field, several other important questions remain open. First, combinatorial effects of multiple modifications on the same transcript are still largely unexplored. Do m^6^A and m^1^A act synergistically or antagonistically in regulating condensate dynamics? Second, the kinetics of writer and eraser enzyme localization to granules under acute as opposed to chronic stress require real-time analysis, potentially through live-cell Nanopore seqencing (Leger et al., 2021) or super-resolution imaging. Third, methyladenine reader proteins beyond the YTH and YBX families might be involved in interpretation of m^1^A and m^6^A within biocondensates and should be systemically identified using proximity-labelling and crosslinking approaches. Fourth, elucidating how these modifications reshape RNA structure *in situ* and modulate the material properties of granules and aggregates will require advanced techniques such as cryo-electron microscopy (cryo-EM) and single-molecule FRET in future studies.

Therapeutically, manipulating RNA modifications offers a promising avenue to modulate condensate behavior in neurodegenerative and other protein-aggregation diseases. For example, small-molecule inhibitors of TRMT61A or activators of ALKBH3 could mitigate pathological m^1^A-dependent TDP-43 aggregation in CAG repeat disorders (Sun et al., 2023). Likewise, allosteric regulators of METTL3 or FTO might adjust m^6^A levels to restore adaptive stress granule dynamics without impairing normal mRNA metabolism. Finally, CRISPR-based epitranscriptomic editing tools could enable locus-specific installation or removal of marks to dissect causality and develop precision therapeutics. As our toolbox expands, combining high-resolution mapping, engineered writer/eraser recruitment, and phase separation biosensors, the field is poised to translate fundamental insights into interventions that restore cellular homeostasis by fine-tuning the epitranscriptomic regulation of phase transitions.

In summary, RNA modifications have emerged as powerful regulators of biomolecular condensates, influencing both physiological granule formation and pathological aggregation. The reversible deposition of m^6^A and m^1^A on mRNA fine-tunes multivalent RNA-protein interactions to either promote dynamic liquid-liquid phase separation or, under dysregulated conditions, drive irreversible fibrillar assemblies. Specifically, m^6^A facilitates stress granule assembly through the multivalent recruitment of YTHDF readers, although its overall contribution to endogenous granule partitioning may be context-dependent (Ries et al., 2019; Khong et al., 2022). On the other side, m^1^A acts as a protective mark in stress granules, facilitating mRNA triage and translational recovery, while on pathological CAG repeat RNAs it aberrantly recruits TDP-43 into neurotoxic inclusions (Alriquet et al., 2020; Sun et al., 2023). Collectively, these modifications appear to constitute an epitranscriptomic code that enables cells to dynamically regulate ribonucleoprotein condensates under stress and thereby preserve proteostasis.

## Abbreviations

ALS, amyotrophic lateral sclerosis; EM, Electron Microscopy; FRET, Förster resonance energy transfer; FTD, frontotemporal dementia; IDR, intrinsically disordered regions; LCD, low-complexity domains; LC-MS/MS, liquid chromatography-tandem mass spectrometry; LLPS, liquid-liquid phase separation; lncRNA, long non-coding RNA; m^1^A, N1-methyladenine; m^6^A, N6-methyladenine; nSB, nuclear stress body; PB, processing bodies; RBP, RNA-binding protein; RRM, RNA recognition motif; SG, stress granules; UTR, untranslated region.
